# A case report of primary ciliary dyskinesia, laterality defects and developmental delay caused by the co-existence of a single gene and chromosome disorder

**DOI:** 10.1186/s12881-015-0192-z

**Published:** 2015-06-30

**Authors:** Jillian P. Casey, Patricia Goggin, Jennifer McDaid, Martin White, Sean Ennis, David R. Betts, Jane S. Lucas, Basil Elnazir, Sally Ann Lynch

**Affiliations:** Genetics Department, Temple Street Children’s University Hospital, Dublin 1, Ireland; UCD Academic Centre on Rare Diseases, School of Medicine and Medical Sciences, University College Dublin, Belfield, Dublin 4, Ireland; Primary Ciliary Dyskinesia Centre, University of Southampton and University Hospital Southampton NHS Foundation Trust, Southampton, UK; National Centre for Medical Genetics, Our Lady’s Children’s Hospital, Crumlin, Dublin 12, Ireland; Neonatology, Our Lady’s Children’s Hospital, Crumlin, Dublin 12, Ireland; Pediatric Respiratory Medicine, The Adelaide and Meath Hospital, Tallaght, Dublin 24, Ireland

**Keywords:** Primary ciliary dyskinesia, Laterality defects, Developmental delay, Single gene disorder, Chromosome disorder, Microduplication syndrome

## Abstract

**Background:**

Primary ciliary dyskinesia (PCD) is a rare autosomal recessive disorder characterised by abnormal ciliary motion and impaired mucociliary clearance, leading to recurrent respiratory infections, sinusitis, otitis media and male infertility. Some patients also have laterality defects. We recently reported the identification of three disease-causing PCD genes in the Irish Traveller population; *RSPH4A*, *DYX1C1* and *CCNO*. We have since assessed an additional Irish Traveller family with a complex phenotype involving PCD who did not have any of the previously identified PCD mutations.

**Case presentation:**

In this study we report on a family with three children with PCD and various laterality defects. In addition, one child (V:1) has mild-to-moderate developmental delay and one child has speech delay (V:2). Developmental delay is not usually associated with PCD and is likely to be caused by an additional genetic abnormality. Transmission electron microscopy showed variable inner and outer dynein arm defects. Exome sequencing identified a homozygous missense variant in *CCDC103* (c.461A > C; p.His154Pro) as the most likely cause of the PCD and laterality defects in this family. However, as mutation in *CCDC103* would not account for the developmental delay, array comparative genomic hybridisation was undertaken and identified a maternally inherited gain of ~1.6 Mb (chr17:34,611,352-36,248,918). Gains at this locus are associated with 17q12 duplication syndrome which includes speech and language delay.

**Conclusion:**

We report on a variable and complex phenotype caused by the co-inheritance of a single gene mutation in *CCDC103* and a microduplication at 17q12, both on chromosome 17. The co-existence of a single gene and chromosome disorder is unusual but accounts for the spectrum of clinical features in this family. In addition, our study brings the total number of PCD genes in the Irish Traveller population to four and we suspect additional PCD genes are yet to be identified. Although, on a global scale, PCD is associated with extensive genetic heterogeneity, finding such a high number of causative PCD genes within the relatively small Irish Traveller population was unexpected.

**Electronic supplementary material:**

The online version of this article (doi:10.1186/s12881-015-0192-z) contains supplementary material, which is available to authorized users.

## Background

Primary ciliary dyskinesia (PCD) is a rare, genetically heterogeneous respiratory disorder primarily characterised by chronic sinopulmonary infections. Approximately 50 % of patients with PCD also have an organ laterality defect. The disorder occurs due to a defect in ciliary structure and/or function. To date, 29 PCD genes have been identified (accounting for ~60 % of cases), 20 of which can cause PCD with a laterality defect [1].

We recently reported the identification of pathogenic variants in three different disease genes causing PCD in the Irish Traveller population; *RSPH4A* (c.166dup; p.Arg56Profs*11), *DYX1C1* (~3.5 kb deletion) and *CCNO* (c.258_262dup; p.Gln88Argfs*8) [1]. Each family had a different type of ultrastructural ciliary defect, depending on which gene was mutated. All patients had normal situs. However, the *DYX1C1* deletion has been observed to cause PCD with laterality defects in an Irish Traveller family (personal communication, Dr. Jane S Lucas). The identification of three PCD genes within a population of ~30,000-40,000 people was surprising and suggests that there may be a selective advantage towards variation in ciliary genes.

**Fig. 1:**
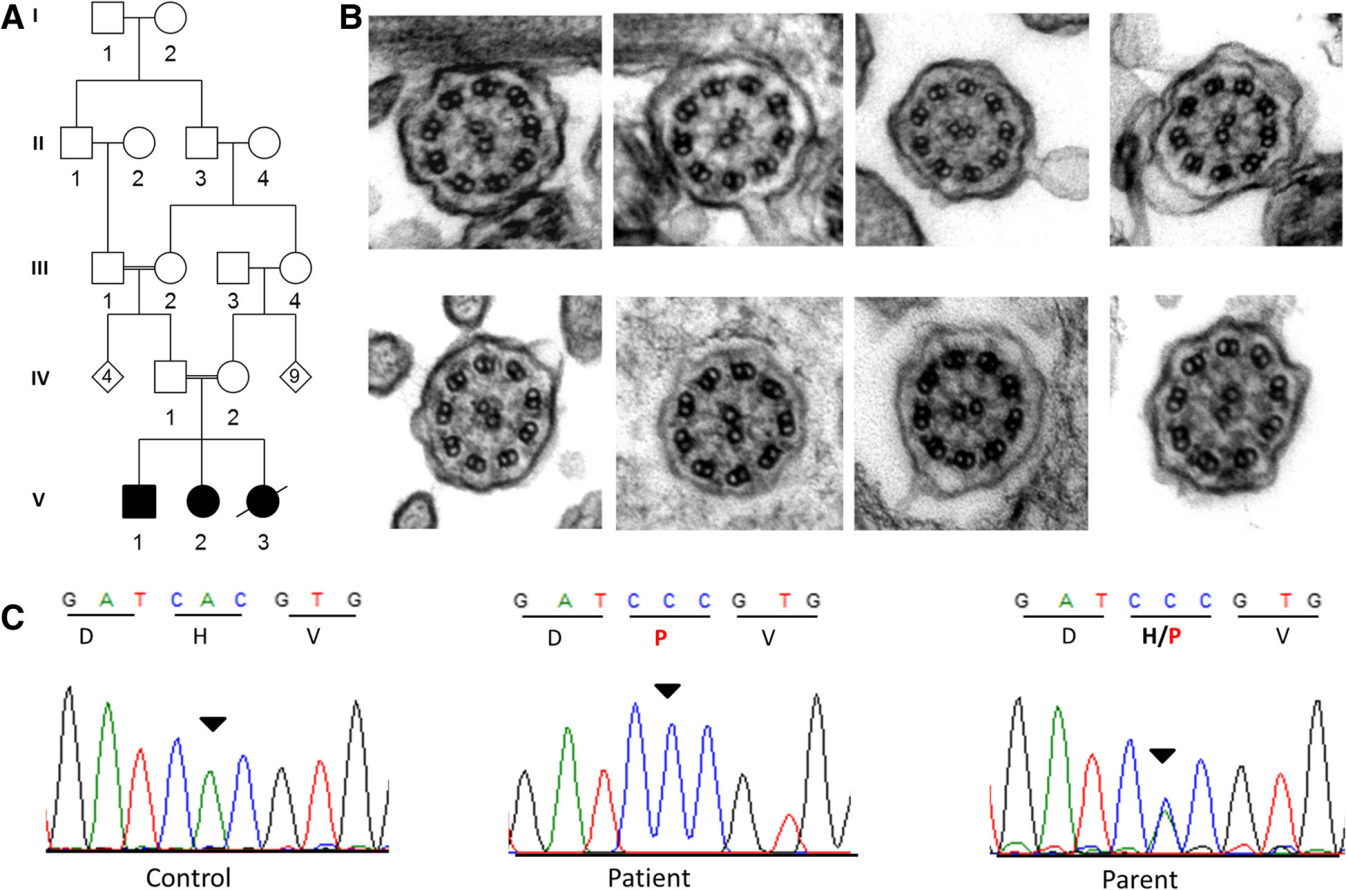
Irish Traveller family with PCD and laterality defects. **a** The family has three children with PCD and various laterality defects. Child V:3 was born prematurely and died post-delivery. DNA was available from all family members. **b** Transmission electron microscopy of bronchial epithelium samples from patient V:2 (1–4) and patient V:1 (5–8) showed a reduced number of dynein arms in some but not all cilia. The phenotype varied from normal ultrastructure (7) through shortened dynein arms (5, arrow) to complete absence of dynein arms. **c** The *CCDC103* NM_001258395.1:c.461A > C was validated by Sanger sequence analysis. The inverted triangle indicates the position of the mutated A > C base on the forward strand which results in the substitution of His (H) with Pro (P) at residue 154

We have since assessed another Irish Traveller family who have three children with PCD and variable laterality defects (Fig. 1a). One child has situs inversus (V:1), one has situs inversus totalis (V:2) and one child (V:3) has left atrial isomerism and an atrioventricular septal defect (AVSD). Sanger sequence analysis excluded the three previously identified PCD mutations in the Irish Traveller population, suggesting that the family harboured a novel mutation. We proposed to identify the underlying genetic cause of PCD with laterality in this family using whole exome sequencing.

## Case presentation

### Sample collection

Genomic DNA was extracted from peripheral blood lymphocytes of the 3 affected children and unaffected parents. The study protocol was approved by the ethics committee of Temple Street Children’s University Hospital (Dublin, Ireland).

### Patient V:1

Patient V:1 was well at birth (no neonatal respiratory distress) but has a history of multi-trigger persistent wheeze, recurrent respiratory tract and ear infections, persistent nasal congestion and possible obstructive sleep apnoea. He also had congenital cytomegalovirus infection which may or may not be linked to moderate bianural hearing loss. He had otitis media with effusion on the left side and Eustachian tube dysfunction on the right side. He has had grommets inserted twice and has had his tonsils and adenoids removed. Sweat test and immunoglobin profiles were normal. A diagnosis of PCD with situs inversus was made. In addition, patient V:1 has mild-to-moderate developmental delay and a pectus excavatum chest deformity. Cardiovascular exam was normal. Chest x-ray was normal with no evidence of dextrocardia. He had brain stem evoked responses which were normal after grommet insertion. Despite normal hearing, his speech is still limited to single words at age 5 years. His gross and fine motor skills are within normal limits. He has mild behavioural issues including bruxism and hyperactivity.

### Patient V:2

Patient V:2 is the only surviving child from a triplet pregnancy. The other two triplets miscarried at 16 weeks gestation. This child was well at birth (no respiratory distress) but she presented with recurrent chest infections as a child and she currently has a wet productive cough and rhinitis. She does not have sinusitis. She has had grommets inserted for otitis media and is awaiting removal of her tonsils and adenoids. Situs inversus totalis was incidentally detected on chest X-ray. She has a structurally normal heart. She has some speech delay.

### Patient V:3

A fetal echocardiogram of patient V:3 showed that she had left atrial isomerism. Antenatal scans did not identify any other internal malformations. She was born prematurely at 26 + 3 weeks gestation following placental abruption and weighed 1.48 kg at birth. Apgar scores were one at 1 min and zero at 5 and 10 min. Full resuscitation, including cardiopulmonary resuscitation, was unsuccessful and she died. There was an antenatal diagnosis of a significant AVSD with hypoplastic right sided tissue. This lesion would not have been treatable unless the baby was born after 32 weeks gestation. She was also dysmorphic with low set ears and broad spaced nipples. Chromosome analysis showed a normal 46XX karyotype. Post mortem was not performed.

### Transmission electron microscopy

Nasal brushings from patients V:1 and V:2 were analysed at a PCD Diagnostic Service (University Hospital Southampton NHS Foundation Trust) following a standard transmission electron microscopy (TEM) protocol [2].

### Whole exome sequencing

DNA from two affected children (V:1 and V:2) was selected for whole exome sequencing. The exonic DNA was enriched with the SureSelect v5 Human All Exon Kit (Agilent Technologies, Santa Clara), and sequenced on an Illumina HiSeq 2500 (Aros Applied Biotechnology, Denmark, now part of the Eurofins Genomics Organisation). The 100 bp paired-end reads were aligned to the hg19 human reference genome. Quality control and variant and indel identification were performed as previously described [1]. Assuming an autosomal recessive model, we prioritised variants that were (i) autosomal, (ii) absent or present with a frequency <1 % in dbSNP130, NHLBI Exome Variant Server database and 1000 Genomes, (iii) homozygous, (iv) absent in our 60 Irish control exomes and (v) shared by the affected siblings.

### Array comparative genomic hybridisation (CGH)

Array CGH was performed using the Agilent design 028469 (~60,000 oligonucleotide probes), with a median resolution of 120 kb. Genomic copy number variants were analysed with Cytogenomics v2.7.22 software (Agilent).

### Validation and segregation

Validation and segregation analysis of the *CCDC103* NM_001258395.1 c.461A>C variant was undertaken by polymerase chain reaction and Sanger sequencing (Additional file 1: Table S1).

### Findings

TEM in patients V:1 and V:2 showed a high percentage of outer and inner dynein arm defects (Fig. 1b). The proportion of cilia with dynein arm defects was not within the range usually seen in patients with PCD but considerably higher than normal (Additional file 1: Table S2). Further investigation was therefore recommended following this inconclusive result. Whole exome sequencing was pursued.

Analysis of the exome data identified 4 rare homozygous variants shared by both siblings, only one of which is in a gene related to ciliary function; *CCDC103* (Additional file 1: Table S3 and S4). *CCDC103* is an essential gene for dynein arm assembly, cilia motility and determination of left-right asymmetry. The *CCDC103* c.461A > C p.His154Pro variant (rs145457535) identified in this family has previously been associated with PCD, varying degrees of situs abnormalities (in some cases) and dextrocardia [3, 4]. Sanger sequencing confirmed that all three affected children are homozygous for *CCDC103* c.461A > C and the parents are obligate carriers (Fig. 1c).

**Fig. 2:**
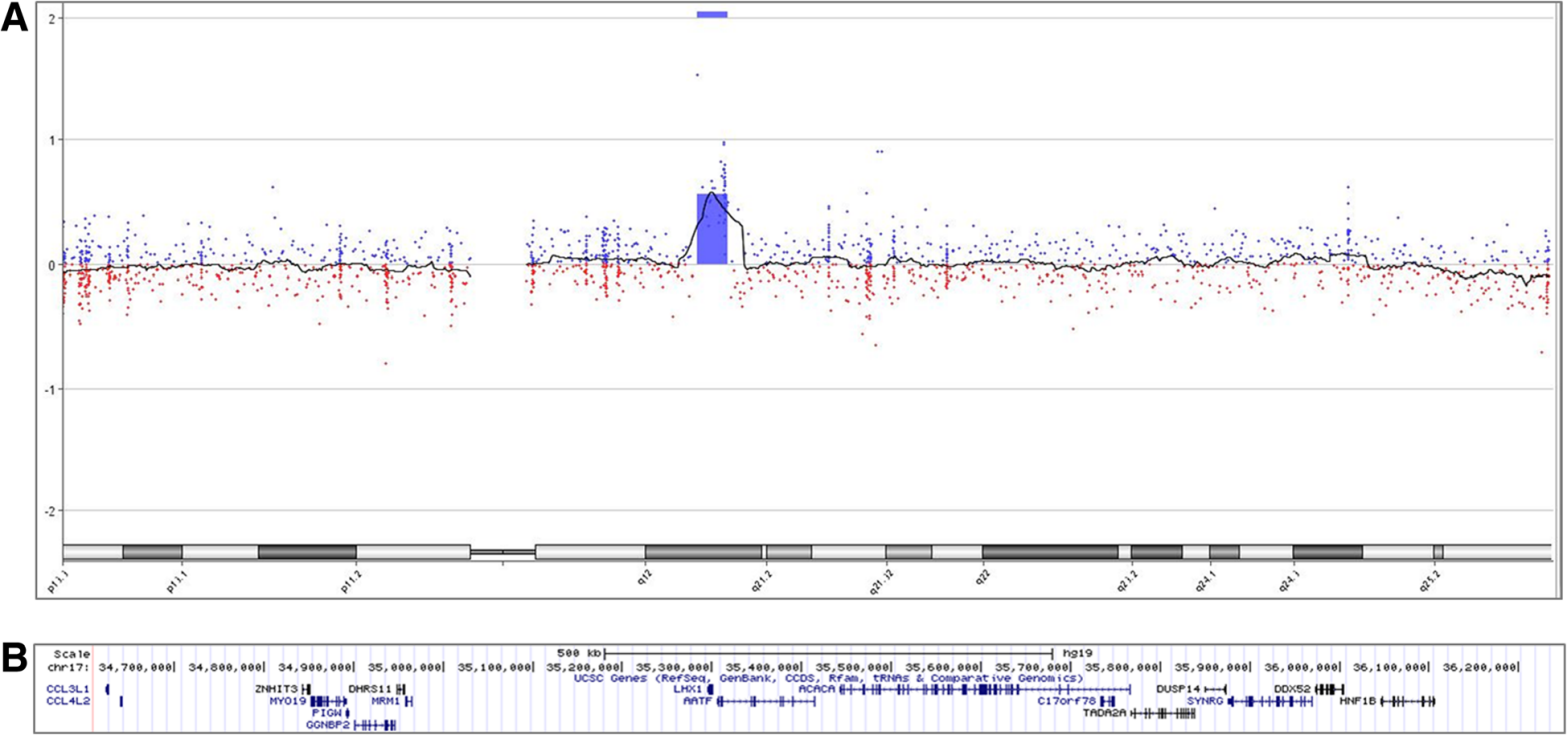
Array comparative genomic hybridisation. Array CGH was undertaken for patient V:1 on account of his developmental delay. **a** Image from Cytogenomics v2.7.22 software (Agilent) showing a ~1.6 Mb gain on 17q12 (hg19; chr17:34,611,352-36,248,918). **b** Screenshot from UCSC hg19 showing the 17 genes (UCSC) located within the duplicated region

Array CGH was performed on account of the developmental and speech delay in patients V:1 and V:2 and identified a gain of ~1.6 Mb at chr17:34,611,352-36,248,918 (Fig. 2). Gains at this locus are associated with 17q12 duplication syndrome (#614526) which includes speech and language delay. Analysis of parental DNA showed that the micro-duplication was maternally inherited. The mother (IV:2) has significant hearing loss due to mastoiditis secondary to chronic ear infection. In her early history, she did not talk until 5 years of age. She was also diagnosed with a goitre and hyperthyroidism as an adult.

## Conclusion

The present study involves three siblings from an Irish Traveller family with PCD, laterality defects, dextrocardia (one child) and developmental delay (two children).

Analysis of ciliary ultrastructure and beat has only been reported for four patients with *CCDC103* mutations; two previously reported and two in the current study (Table 1). The patients reported in this study showed reduced numbers of dynein arms on TEM in comparison to healthy controls, but not as low as typically found in PCD. Whole exome sequencing identified *CCDC103* c.461A > C p.His154Pro as the most likely cause of the PCD and laterality phenotype in this family. This finding brings the number of PCD genes in the relatively small Irish Traveller population to four, highlighting locus heterogeneity even in a small endogamous population. Given the rarity of PCD (1:15,000-30,000), finding such a high number of causative genes in a genetically homogenous endogamous population was unexpected.

**Table 1:** Clinical and histological characterisation of patients homozygous for *CCDC103* p.His154Pro

Patient	ODA	IDA	Motility	PCD	Laterality	Other	Origin	Cons	Ref
UCL-143	Complete	Complete	n.a.	+	Normal		Pakistani	yes	3
II1	loss	loss				-			
OP-1194	n.a	n.a	n.a.	+	Dextrocardia		Pakistani	yes	3
II1						-			
OP-32	Reduction of ODAs	No defect reported	Reduced cilia beat amplitude	+	SI totalis		German	no	3
II1						-			
OP-32	n.a.	n.a	Loss of beat coordination, and cilia paralysis	+	SI abdominalis		German	no	3
II2				+		-	Italian	no	4
D’Andrea et al. II-2	n.a	n.a	n.a.	+	SI totalis	AVSDPFO	Italian	no	4
D’Andrea et al. II-3	n.a	n.a	n.a.	+	SI totalis	AVSDOS	Irish Traveller	yes	c.s.
Casey et al. V:1	Reduction in ODAs	Reduction in IDAs	n.d.	+	SI	-	Irish Traveller	yes	c.s.
Casey et al. V:2	Reduction in ODAs	Reduction in IDAs	n.d.	+	SI totalis	-			
Casey et al. V:3	n.d.	n.d.	n.d.		Left atrial isomerism	AVSD	Irish Traveller	yes	c.s.

Features that are present and absent are denoted by + and – respectively. *Abbreviations: AVSD* atrioventricular septal defect, *Cons* consanguinity, *c.s.* current study, *IDA* inner dynein arm defects, *n.a* not available, *n.d.* not done, *ODA* outer dynein arm defects, *OS* ostium secundum, *PCD* primary ciliary dyskinesia, *PFO* patent foramen ovale, *Ref* reference, *SI* situs inversus, *SI totalis* situs inversus including dextrocardia

The *CCDC103* p.His154Pro variant has previously been associated with PCD and laterality defects in families from three other populations; two Pakistani families, one German family and one Italian family [3, 4]. We now report the presence of this particular variant in the Irish Traveller population. Identification of the same variant across multiple populations supports the causative nature of the p.His154Pro variant and suggests either a founder mutation or a mutation hotspot. Given the absence of any known shared ancestry between the three populations, this observation is more likely due to a spontaneous mutation hotspot at cDNA position 461.

Almost 50 % of PCD patients have situs inversus totalis, while at least 12 % have incomplete situs inversus or heterotaxy [5]. A total of nine PCD patients from five families (including current study) have been reported to be homozygous for the *CCDC103* p.His154Pro variant (Table 1). Laterality status ranges from situs solitus (normal) to situs inversus totalis. This variation in laterality may be due to a combination of timing and the suggested hypomorphic nature of the p.His154Pro variant. The timing of alterations during the patterning process has a profound effect on the final laterality phenotype [6]. It is possible that the p.His154Pro variant may result in varying levels of protein of reduced functionality; patients with the least amount of functional protein during the earliest steps in the establishment of left-right asymmetry may develop situs inversus totalis, whereas those retaining greater amounts of functional protein may develop situs inversus abdominalis or have normal situs. Panizzi and colleagues also observed considerable phenotypic variation in a zebrafish p.His154Pro model, suggesting the influence of modifier genes [3].

Of the three affected siblings with the *CCDC103* p.His154Pro variant reported in the current study, only one child (V:3) (with left isomerism) has an AVSD. Two of the six previously reported patients with the p.His154Pro variant also had an AVSD (Table 1). Congenital heart defects are not uncommon in patients with lateralisation disorders as laterality disturbance can affect heart development. AVSD associated with laterality defects occurs in approximately 91.2 % of right isomerism compared to 56.8 % of left isomerism cases [7]. Genes causative of AVSD have demonstrated incomplete penetrance and variable expression which correlates with the presence of AVSD in 3/9 *CCDC103* p.His154Pro patients to date.

**Fig. 3:**
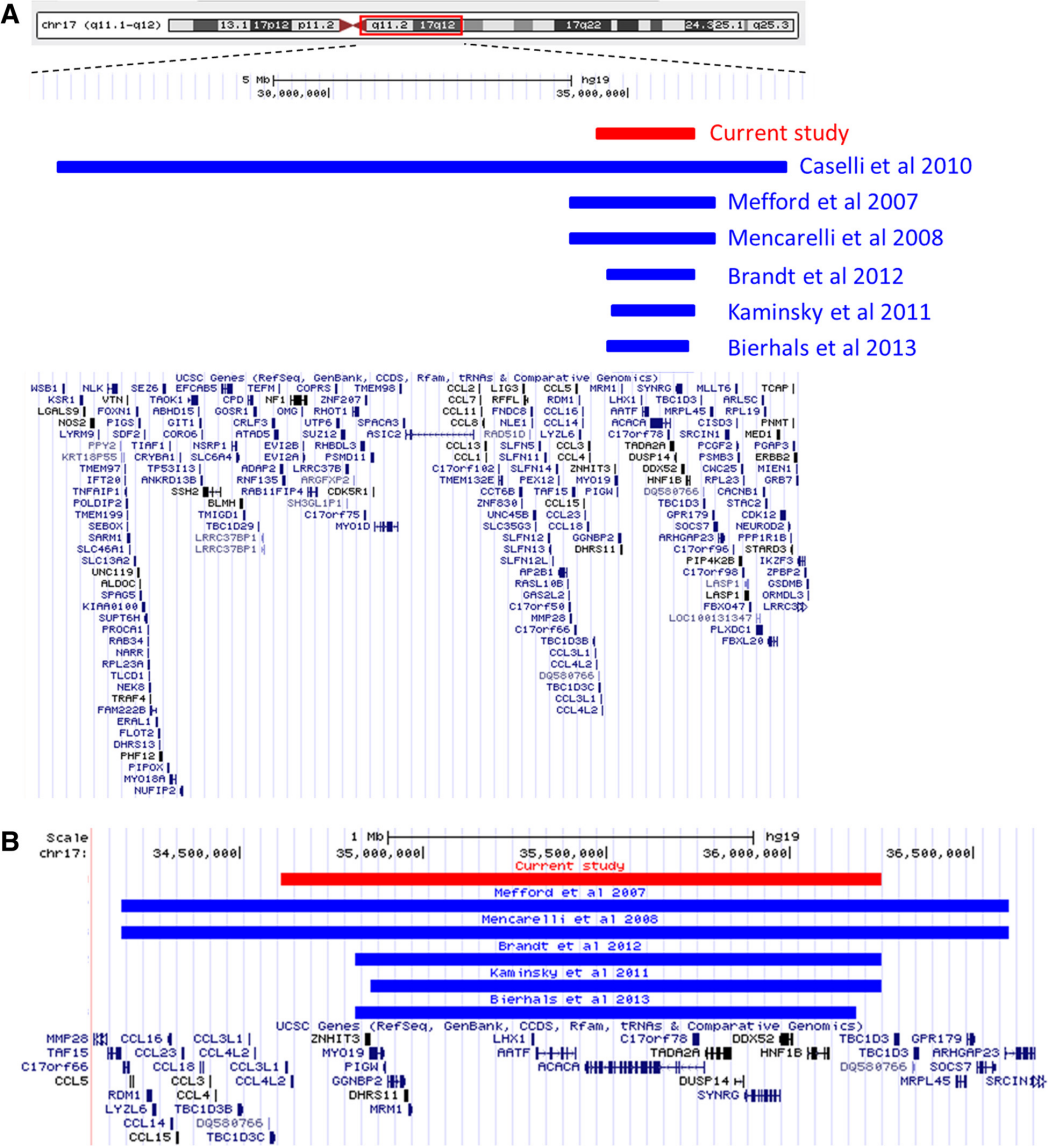
17q12 microduplications. **a** UCSC Genome Browser (hg19) view of previously reported 17q12 microduplications and one large 12.4 Mb duplication. The chromosomal region 17q11.2-q12 is shown together with UCSC genes. The coloured bars show the location and extent of the duplicated region of the patients described here (red), and of other cases reported in the literature (blue). **b** Zoomed in view of genes within the microduplication reported in this study (red) and previously reported 17q12 microduplications (blue). Duplications range in size from 300 kb to 2.4 Mb

While the *CCDC103* variant explains the PCD, laterality defects and AVSD in this family, it is unlikely to account for the developmental delay. Array CGH analysis showed that in addition to the *CCDC103* gene mutation, at least two of the children have a maternally inherited 17q12 microduplication of approximately 1.6 Mb. This 17q12 microdupliation overlaps with duplications previously reported in other patients (Fig. 3a). There is one known disease gene, *HNF1B (TCF2)*, located within the critical region (Fig. 3b). Deletion of *HNF1B* is associated with renal cysts and maturity-onset diabetes type 5 while there is one report of a *HNF1B* duplication causing various renal abnormalities [8, 9]. As the 17q12 duplication identified in the current study includes this gene, it is possible that the patients may be at risk of developing renal cysts later in life, though this is an ultrarare feature of the duplication syndrome. Furthermore, 17q12 duplications are associated with a wide phenotypic spectrum, even in patients with the exact same breakpoints which complicates genotype-phenotype correlation [10].

Patients carrying a 17q12 duplication present with a variable phenotype characterised by intellectual disability or developmental delay of various degree. Additional features may include behavioural problems, brain abnormalities, epilepsy, esophageal atresia, renal abnormalities, atrial septal defects, sex reversal and ocular anomalies [11–15]. A 17q12 microduplication has also been identified in patients with autism spectrum disorder [16]. The patients in the present study presented with mild-to-moderate developmental delay only. The mother (IV:2), who is the carrier of the microduplication, did not talk until age 5, consistent with previously reported features of 17q12 microduplcation. Array CGH was not performed on DNA from the deceased child (V:3). However, it is plausible to suggest that she too had the 17q12 microduplication as she had facial dysmorphism and widely spaced nipples, which have been reported in patients with 17q12 anomalies. Of note, there is one previous report of a child with PCD, dextrocardia, AVSD, mild facial dysmorphism and wide spaced nipples [17]. However, the molecular basis of the child’s disorder was not reported.

In terms of genetic counselling, it is important to try and establish which clinical features are attributable to each of the *CCDC103* mutation and the 17q12 microduplication. It is clear that the PCD, recurrent infections and laterality defects are a direct result of the *CCDC103* mutation. Although it is not uncommon for PCD patients to have associated speech and language delay due to conductive hearing loss caused by recurrent chronic otitis media and effusions [18], we do not think this is the case in the reported family. Brain stem evoked responses were normal after grommet insertion in patient V:1. Despite normal hearing, his speech is still limited to single words (no sentences) by age 5 years. Therefore, the speech and language delay together with his behavioural issues are most likely attributable to the 17q12 microduplication. While his sister (V:2) has only mild speech delay, this variability is frequently found in patients with 17q12 microduplications whereby development can range from normal to severely delayed.

**Fig. 4:**
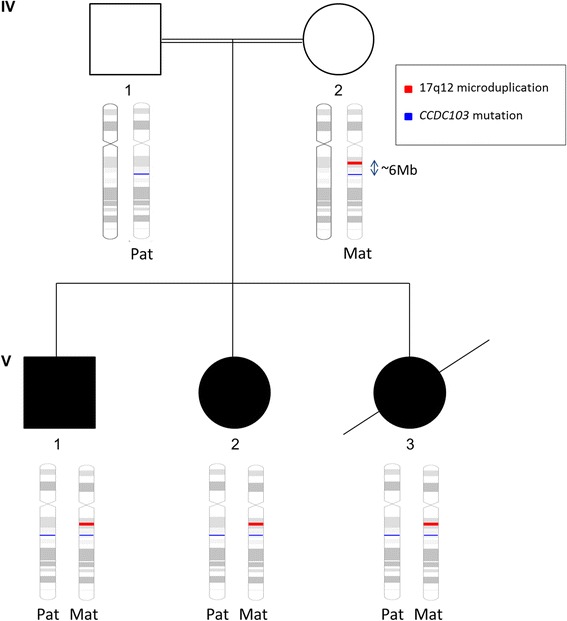
Co-inheritance of two genetic alterations. The *CCDC103* variant (blue) and the 17q12 microduplication (red) are both located on chromosome 17, approximately 6 Mb apart. Given the co-inheritance of PCD/laterality with the 17q12 microduplication syndrome, the mother (IV:2) most likely carries the two genetic alterations in cis. The father (IV:1) carries only the *CCDC103* variant. Therefore, all offspring within this family who have PCD and laterality defects will also have 17q12 microduplication syndrome, provided there is no recombination on the maternal chromosome

It is interesting to note that the *CCDC103* variant and the microduplication are both located on chromosome 17. We do not believe that one genetic alteration “caused” the other but it suggests co-inheritance. The mother (IV:2) carries both the 17q12 microduplication and the *CCDC103* variant. At least two of her children have inherited both the microduplication and the *CCDC103* variant, indicating that these two alterations are in cis. The father (IV:1) carries the *CCDC103* variant only. Therefore, any child in this family who inherits the *CCDC103* variant from their mother, will also inherit the microduplication provided there is no recombination (Fig. 4). This suggests that any future children in this family who present with PCD and/or laterality should also be tested for the microduplication which is likely to be co-inherited. Given that the parents are first cousins (mothers are sisters), the *CCDC103* variant carried by each parent (IV:1 and IV:2) is likely the same mutation inherited from a common ancestor. The DNA surrounding the *CCDC103* variant should therefore be the same on both the mother’s and father’s chromosome 17. However, the fact that the father does not carry the microduplication suggests that this copy number alteration arose recently as a *de novo* evolutionary event in either the carrier mother (IV:2) or in her mother (III:4) (who is the maternal aunt of her husband). These observations suggest that there are two *CCDC103* “alleles” in the Traveller population; one which contains only the *CCDC103* variant and another which contains both the *CCDC103* variant and the 17q12 microduplication. Hence, it is possible for some Traveller patients to have isolated PCD/laterality due to the *CCDC103* alteration while others will be at risk of the microduplication syndrome depending on which “allele” they inherit.

In conclusion, we report a rare finding of patients with both a single gene disorder (*CCDC103*) and a chromosome disorder (17q12 microduplication) giving rise to a complex and variable phenotype. Together, the two genetic alterations account for the spectrum of clinical features observed in the family. Our findings highlight the importance of considering the possibility of more than one genetic mutation in patients with a complex phenotype, which cannot be explained by abnormalities in one gene alone.

### Consent

We have obtained written informed consent from the parents of the patients for publication of this case report and any accompanying images. A copy of the written consent is available for review from the Editor of this journal.

## Additional file

Additional file 1: Table S1. Primer sequences for PCR amplification. **Table S2.** Transmission electron microscopy. **Table S3.** Variant prioritisation strategy. **Table S4.** Rare homozygous variants shared by siblings.

## Abbreviations

PCD: Primary ciliary dyskinesia
AVSD: Atrioventricular septal defect
TEM: Transmission electron microscopy
CGH: Comparative genomic hybridisation
