# Supplementary material for: A case report of primary ciliary dyskinesia, laterality defects and developmental delay caused by the co-existence of a single gene and chromosome disorder
Source: BMC Med Genet. 2015 Jun 30;16:45. doi: 10.1186/s12881-015-0192-z (PMC4630905; doi:10.1186/s12881-015-0192-z)
Supplement: Additional file 1: Table S1. — Primer sequences for PCR amplification. Table S2. Transmission electron microscopy. Table S3. Variant prioritisation strategy. Table S4. Rare homozygous variants shared by siblings. [file 12881_2015_192_MOESM1_ESM.docx]

**Supplementary Material**

**A case report of primary ciliary dyskinesia, laterality defects and developmental delay caused by the co-existence of a single gene and chromosome disorder**

Jillian P. Casey^1,2^, Patricia Goggin^3^, Jennifer McDaid^4^, Martin White^5^, Sean Ennis^2,5^, David R. Betts^4^, Jane S. Lucas^3^, Basil Elnazir^6^, Sally Ann Lynch^1,2,4^

^1^Genetics Department, Temple Street Children’s University Hospital, Dublin 1, Ireland.^2^UCD Academic Centre on Rare Diseases, School of Medicine and Medical Sciences, University College Dublin, Belfield, Dublin 4, Ireland.^3^Primary Ciliary Dyskinesia Centre, University of Southampton and University Hospital Southampton NHS Foundation Trust, Southampton, UK. ^4^National Centre for Medical Genetics, Our Lady’s Children’s Hospital, Crumlin, Dublin 12, Ireland. ^5^Neonatology, Our Lady’s Children’s Hospital, Crumlin, Dublin 12, Ireland. ^6^Pediatric Respiratory Medicine, The Adelaide and Meath Hospital, Tallaght, Dublin 24, Ireland.

Corresponding author: Dr Jillian Casey (jillian.casey@ucd.ie)

Department of Genetics, Temple Street Children’s University Hospital, Dublin 1, Ireland.

Phone: +353 1 4096298 Fax: +353 1 4560953

**Supplementary Table S1. Primer sequences for PCR amplification**

| **Variant** | **Forward Primer**  **5’-3’** | **Reverse Primer**  **5’-3’** | **Annealing temperature** | **PCR product size (bp)** |
| --- | --- | --- | --- | --- |
| *CCDC103* NM_001258395.1:c.461A>C | aagagctacaggctcccctc | ggaaccaggtgtgggtttc | 58°C | 641 |

The *CCDC103* candidate variant was validated by polymerase chain reaction and Sanger sequence analysis. DNA from available family members was also analysed by Sanger sequencing to test for segregation.

**Supplementary Table S2. Transmission electron microscopy**

| **Cross section details** | **V:1** | **V:2** |
| --- | --- | --- |
| Number of cilia counted | 285 | 102 |
| Normal microtubule pattern | 94.75% | 75.5% |
| Disarranged outer microtubules | 1.75% | 1.96% |
| Extra tubule | 0.35% | 3.92% |
| Single tubule | 0% | 0% |
| Transposed tubules | 0% | 0% |
| Central pair; one tubule missing | 1.05% | 0% |
| Central pair; both tubules missing | 1.05% | 2.94% |
| Compound | 0.70% | 2.94% |
| Outer dynein arms missing | 2.94% | 5.13% |
| Inner dynein arms missing | 11.76% | 17.95% |
| Both inner and outer arms missing | 64% | 33.33% |

Nasal brushings from two of the affected siblings were analysed by transmission electron microscopy and showed both inner and outer dynein arm defects. Defects were less numerous than expected for PCD, but higher than that observed in a normal sample.

**Supplementary Table S3. Variant prioritisation strategy**

| **Prioritisation Parameter** | **V:2** | **V:1** |
| --- | --- | --- |
| Autosomal coding variants which are absent or present with a frequency <1% in dbSNP, NHLBI ESP and 1000G | 490 | 546 |
| + Homozygous | 62 | 44 |
| + Absent or present with a frequency <1% in Irish control exomes | 18 | 8 |
| + shared by both affected siblings | **4** | |

Assuming an autosomal recessive model, we prioritised coding (missense, nonsense, splice site and indels) variants that were (i) autosomal, (ii) absent or present with a frequency <1% in dbSNP130, NHLBI Exome Variant Server database and 1000 Genomes, (iii) homozygous, (iv) absent or present with a frequency <1% in our 60 Irish control exomes, and (v) shared by the affected siblings for whom exome analysis was undertaken.

**Supplementary Table S4. Rare homozygous variants shared by siblings**

| **Gene** | **Transcript** | **Change at cDNA level** | **Change at protein level** | **dbSNP ID** | **MAF** |
| --- | --- | --- | --- | --- | --- |
| *HES6* | NM_018645.5 | c.559insG | p.E187fs | No ID | 0.02% |
| *CDH23* | NM_022124.5 | c.3118G>T | p.D1040Y | rs200177873 | 0.01% |
| *LINC00452* | NM_001278674.1 | c.742C>T | p.R248C | rs140065392 | 0.32% |
| *CCDC103* | NM_001258395.1 | c.461A>C | p.H154P | rs145457535 | 0.10% |

Whole exome sequencing and variant prioritisation identified 4 novel or rare homozygous candidate variants that were shared by two of the affected siblings (V:1 and V:2).
